# Gender Differences in Cardiogenic Shock Patients: Clinical Features, Risk Prediction, and Outcomes in a Hub Center

**DOI:** 10.3389/fcvm.2022.912802

**Published:** 2022-07-13

**Authors:** Sara Lozano-Jiménez, Reyes Iranzo-Valero, Javier Segovia-Cubero, Manuel Gómez-Bueno, Mercedes Rivas-Lasarte, Cristina Mitroi, Juan Manuel Escudier-Villa, Juan Francisco Oteo-Dominguez, Jose María Vieitez-Florez, Susana Villar-García, Francisco José Hernández-Pérez

**Affiliations:** ^1^Department of Cardiology, Hospital Puerta de Hierro, Madrid, Spain; ^2^Centre of Biomedical Research in Cardiovascular Diseases (CIBERCV), Carlos III, Madrid, Spain; ^3^Department of Anesthesiology, Hospital Puerta de Hierro, Madrid, Spain; ^4^Department of Cardiovascular Surgery, Hospital Puerta de Hierro, Madrid, Spain

**Keywords:** cardiogenic shock, gender, SCAI classification, heart failure, prognosis, mortality

## Abstract

**Introduction:**

There is scarce knowledge about gender differences in clinical presentation, management, use of risk stratification tools and prognosis in cardiogenic shock (CS) patients.

**Purpose:**

The primary endpoint was to investigate the differences in characteristics, management, and in-hospital mortality according to gender in a cohort of CS patients admitted to a tertiary hub center. The secondary endpoint was to evaluate the prognostic performance of the Society of Cardiovascular Angiography and Interventions (SCAI) classification in predicting in-hospital mortality according to sex.

**Methods:**

This is a retrospective single-Center cohort study of CS patients treated by a multidisciplinary shock team between September 2014 and December 2020. Baseline characteristics and clinical outcomes according to gender were registered. Discrimination of SCAI classification was assessed using the area under the receiver operating characteristic curve (AUC).

**Results:**

Overall, 163 patients were included, 39 of them female (24%). Mean age of the overall cohort was 55 years (44–62), similar between groups. Compared with men, women were less likely to be smokers and the prevalence of COPD and diabetes mellitus was significantly lower in this group (*p* < 0.05). Postcardiotomy (44 vs. 31%) and fulminant myocarditis (13 vs. 2%) were more frequent etiologies in females than in males (*p* = 0.01), whereas acute myocardial infarction was less common among females (13 vs. 33%). Regarding management, the use of temporary mechanical circulatory support, mechanical ventilation, or renal replacement therapy was frequent and no different between the groups (88, 87, and 49%, respectively, in females vs. 42, 91, and 41% in males, *p* > 0.05). In-hospital survival in the overall cohort was 53%, without differences between groups (52% in females vs. 55% in males, *p* = 0.76). Most of the patients (60.7%) were in SCAIE at presentation without differences between sexes. The SCAI classification showed a moderate ability for predicting in-hospital mortality (overall, AUC: 0.653, 95% CI 0.582–0.725). The AUC was 0.636 for women (95% CI 0.491–0.780) and 0.658 for men (95% CI 0.575–0.740).

**Conclusions:**

Only one in four of patients treated at a dedicated CS team were female. This may reflect differences in prevalence of severe heart disease at young (<65) ages, although a patient-selection bias cannot be ruled out. In this very high-risk CS population of multiple etiologies, overall, in-hospital survival was slightly above 50% and showed no differences between sexes. Treatment approaches, procedures, and SCAI risk stratification performance did not show gender disparities among treated patients.

## Introduction

Cardiogenic shock (CS) is a life-threatening condition. In spite of recent advances in its management, morbidity and mortality remains high (1) and only emergency revascularization in CS complicating acute myocardial infraction (AMI) has shown a significant survival benefit (2).

There is scarce knowledge about gender differences in clinical presentation and prognosis of CS. Previous studies in this field are based on AMI-related CS and women tended to have a higher mortality (3–5). However, it has been argued that this fact might be explained by an older age in female patients. Furthermore, results among the different authors are conflicting and there is a lack of consensus on whether gender is associated with outcomes in CS (6).

On the other hand, a classification of the Society of Cardiovascular Angiography and Interventions (SCAI) has been recently proposed. It can be easily obtained at bedside and stratifies CS in 5 stages from least to greatest severity (A: “at risk”; B: “beginning”; C: “Classic”; D: “Deteriorating” or E: “Extremis”) (7). There are insufficient data regarding to gender-associated differences for this risk stratification tool.

Therefore, the primary end-point of this study was to investigate the influence of gender on in-hospital mortality. Secondary end-points were to evaluate differences between gender regarding comorbidities, clinical presentation and treatment approaches for CS. Finally, we analyzed the yield of SCAI classification in both sexes.

## Materials and Methods

### Study Design, Inclusion Criteria and Data Collection

We performed a retrospective observational study of a cohort of CS patients managed by a multidisciplinary team in a hub center between September 2014 and December 2020. It has been considered to be managed in this Unit those patients with refractory cardiogenic shock and/or patients who are candidates for advanced heart failure therapies, such as transplantation or LVAD (8).

Baseline characteristics and clinical outcomes according to gender were registered. Mean age of the overall cohort was 55 years (44–62). CS was defined by a systolic blood pressure < 90 mmHg for more than 30 min or inotropes required to maintain a mean blood pressure > 65 mmHg and signs of impaired organ perfusion with at least one of the following: altered mental status, urine output <30 ml/h or serum lactate > 2 mmol/l. Patients were assigned to one of the five SCAI stages by two independent cardiologists, who were blind to each other's classification. SCAI CS subgroups were interpreted considering the recent consensus statement (7); based on clinical, laboratory, and hemodynamic parameters. In our case, due to the nature of the CS Unit, all the patients fulfilled at least stage C. Most patients were classified as stage D due to clinical and/or biochemical worsening in the first hours, addition of 2 or more vasoactive drugs, and/or need or change of mechanical circulatory support (MCS). Those classified as stage E had combinations of some or all of the following characteristics: cardiac arrest requiring cardiopulmonary resuscitation and/or venoarterial extracorporeal membrane oxygenation (ECMO), mechanical ventilation, profound acidosis (pH < 7.2) and/or lactate > 5 mmol/l, refractory ventricular arrhythmias or sustained hypotension despite maximum support.

The study was approved by Local Institutional Ethics Committee.

### Statistical Analysis

Continuous variables are reported as mean (standard deviation) or median (interquartile range) as appropriate. For categorical variables, frequencies and percentages are presented. Statistical differences were analyzed using T-student (for continuous variables) or the χ^2^ test/fisher's exact test (for categorical variables). Discrimination of the SCAI classification was assessed with the area under the receiver-operating characteristic curve (AUC) and its calibration with the Hosmer-Lemeshow test. Two-tailed *p* < 0.05 were considered statistically significant. All analyses were performed using statistical software STATA IC/13 (Stata corp, College Station, TX, USA).

## Results

### Sex-Differences in Baseline Characteristics of the Study Population

**Figure 1:**
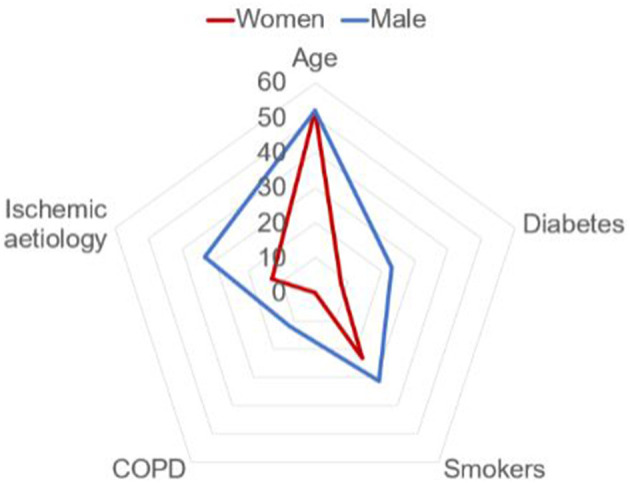
Baseline characteristics according to gender.

Overall, 163 patients were included and 39 of them female (24%). Mean age of the overall cohort was 55 years (44–62), similar between groups. Compared with men, women were less likely to be smokers, and the prevalence of COPD and diabetes mellitus was significantly lower in this group (*p* < 0.05). Postcardiotomy (44 vs. 31%) and fulminant myocarditis (13 vs. 2%) were more frequent etiologies in females than in males, whereas CS was less often related to AMI in women (13 vs. 33%) (Figure 1). However, other relevant characteristics did not differ between both sexes (Table 1).

**Table 1:** Baseline characteristics, treatment, and outcomes according to gender.

		**Male (*n* = 124)**	**Female (*n* = 39)**	***P*-value**
Mean age (years)		52 (44–62)	53 (43–62)	0.91
**Comorbidities:** ***n*** **(%)**				
Hypertension		48 (39)	12 (31)	0.37
Dyslipidemia		44 (35)	8 (21)	0.07
Diabetes mellitus		29 (23)	3 (8)	0.03
Smoking		38 (31)	9 (23)	0.01
Chronic kidney disease		14 (11)	2 (5)	0.25
COPD		15 (12)	0 (0)	0.02
Prior stroke		9 (7)	6 (15)	0.12
Previous heart disease		73 (59)	23 (59)	0.09
**Etiology of CS:** ***n*** **(%)**				
ADHF	Overall	36 (30)	9 (23)	0.01
	- Ischemic	10 (28)	2 (22)	0.01
	- Non ischemic	26 (72)	7 (78)	
Acute myocardial infarction		40 (33)	5 (13)	
Postcardiotomy	Overall	37 (31)	17 (44)	
	- CABG	15 (41)	4 (23)	
	- Valvular heart surgery	12 (32)	8 (47)	
	- Acute MI	0	0	
	- PGD	9 (24)	4 (23)	
	- Other causes	1 (3)	1 (7)	
Myocarditis		3 (2)	5 (13)	
Other causes		8 (6)	3 (8)	
**Clinical presentation:** ***n*** **(%)**				
SCAI C		6 (5)	3 (8)	0.42
SCAI D		45 (36)	10 (26)	
SCAI E		73 (59)	26 (67)	
Prior cardiac arrest		43 (35)	10 (26)	0.29
Mean blood pressure (mmHg)		81 ± 17	73 ± 17	0.86
**Treatment and procedures:** ***n*** **(%)**				
Vasoactive-inotropic score in the first 24 h		44 ± 5	50 ± 10	0.74
Intra-aortic balloon pump		73 (59)	22 (56)	0.78
Mechanical circulatory support (MCS)		99 (80)	33 (85)	0.24
VA-ECMO		52 (42)	24 (62)	
Levitronix Centrimag		30 (24)	7 (18)	
Impella		17 (14)	2 (5)	
Mechanical ventilation		113 (91)	34 (87)	0.47
Renal replacement therapy		51 (41)	19 (49)	0.40
**Outcome**				
Initial mortality due to CS		52 (42%)	17 (43%)	0.88
Recovery		35 (28%)	12 (31%)	
Heart replacement (Heart transplant or LVAD)		37 (30%)	10 (26%)	
Overall in-hospital survival		67 (55%)	20 (52%)	0.76

*ADHF, Acute decompensation of chronic heart failure; CABG, Coronary Artery Bypass Graft surgery; COPD, Chronic obstructive pulmonary disease; CS, Cardiogenic shock; LVAD, Left ventricular assist device; MCS, Mechanical circulatory support; MI, Myocardial infarction; PGD, Primary Graft Dysfunction; SCAI, Society of Cardiovascular Angiography and Interventions; VA- ECMO, venoarterial extracorporeal membrane oxygenation. Categorical variables are expressed as No. (%) and continuous variables as mean ± standard deviation or median (interquartile range)*.

### Management and Outcomes According to Sex

Regarding management, the use of temporary MCS was high and no different between the groups (female 33/39 [84%] vs. male 99/124 [80%]; *p* = 0.24). The vast majority of patients required only one MCS procedure (138/163; 85%). However, 25 patients (15%) needed two or more devices. Most of them were converted to Levitronix Centrimag® (21/25; 84%). Likewise, escalation of MCS did not differ according to gender (13% in female patients vs. 19% in male, *p* = 0.61).

Furthermore, the use of mechanical ventilation (87 vs. 91%) and renal replacement therapy (49 vs. 41%) were not significantly different either.

In-hospital survival rate in the overall cohort was 53%, without differences between the groups (female 20/39 [52%] vs. male 67/124 [55%], *p* = 0.76). Likewise, use of advanced heart failure therapies such as heart transplantation or left ventricular assist device (LVAD) did not differ according to gender (26 vs. 30%, *p* = 0.96).

### Performance of the SCAI Classification in Predicting In-hospital Mortality

No significant differences were observed regarding SCAI classification according to gender (*p* = 0.42), with the highest proportion of patients in SCAI class E (60.7%).

**Figure 2:**
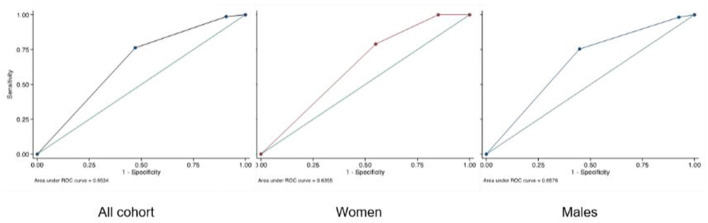
AUC of SCAI classification.

**Figure 3:**
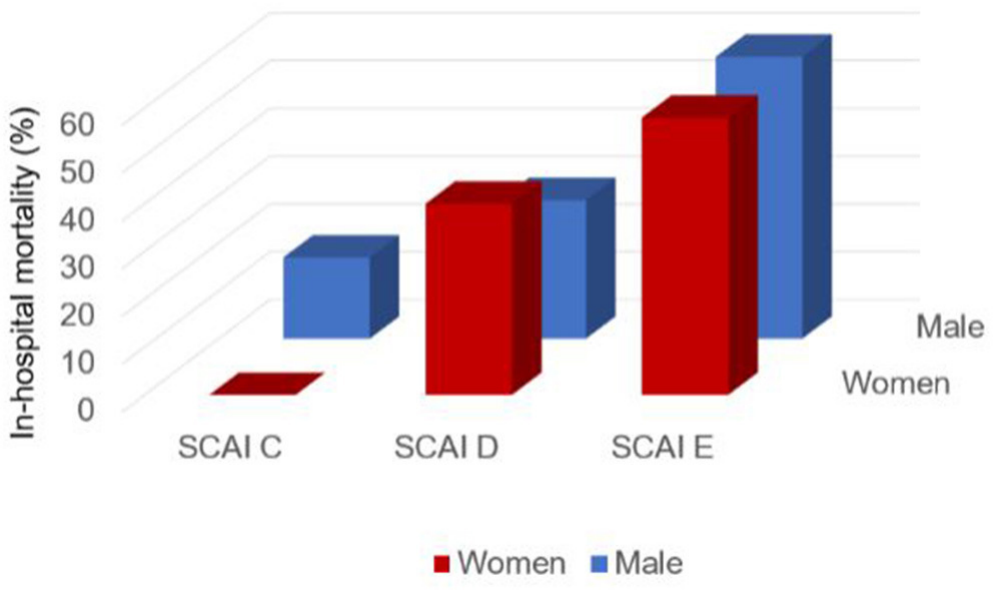
In-hospital mortality according to SCAI and gender.

**Figure 4:**
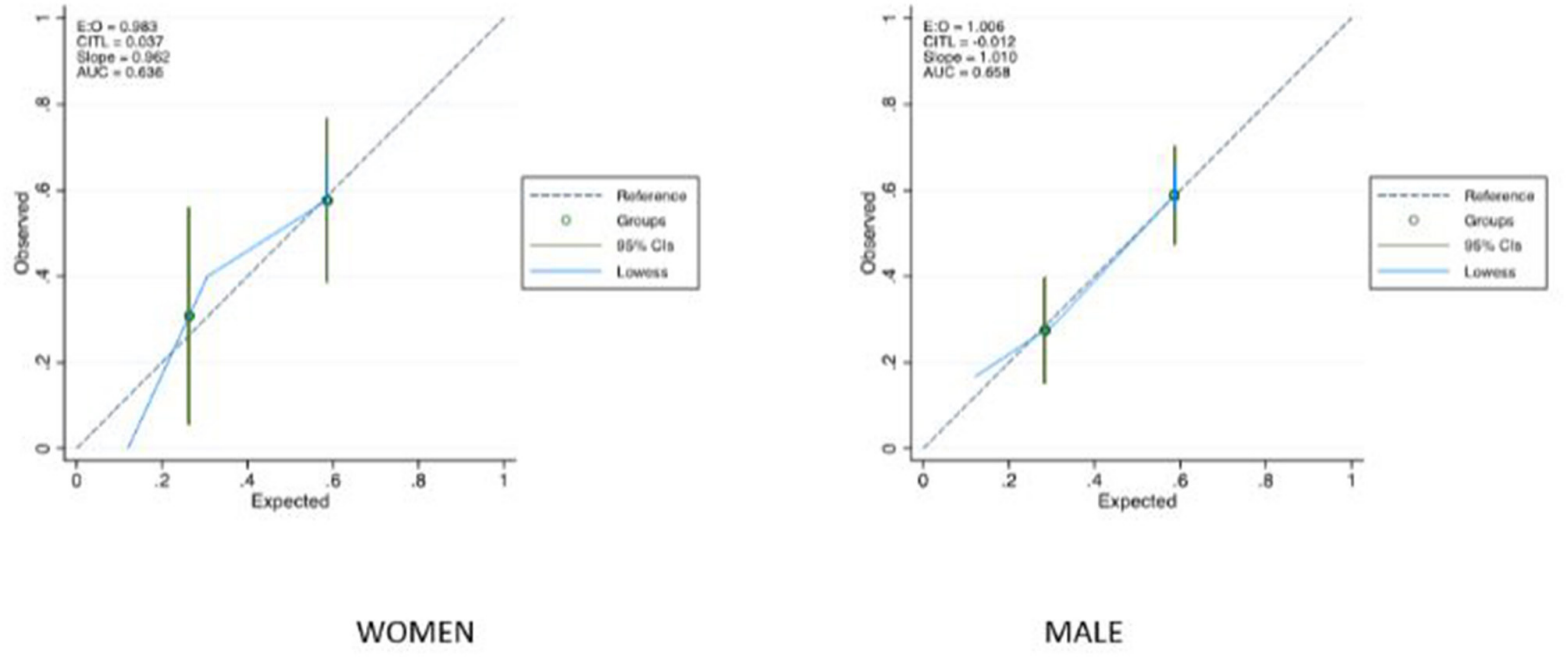
Calibration of SCAI classification.

The SCAI classification showed a moderate ability for predicting in-hospital mortality AUC: 0.653 (95% CI 0.582–0.725). The AUC was 0.636 in women (95% CI 0.491–0.780) and 0.658 in men (95% CI 0.575–0.740) (Figure 2). Figure 3 shows in-hospital mortality in each stage according to gender. It was 0, 25, and 42% in stages C, D, and E respectively in female patients and 13, 26, and 54% in stages C, D and E respectively in male (*p* = 0.06). Calibration of the SCAI classification was good (Hosmer-Lemeshow *p* = 0.92) and similar between sexes (Figure 4).

There were no differences in performance between SCAI categorization in the overall cohort and VIS score. This last stratification tool had an AUC of 0.67 (CI 95% 0.57–0.74).

## Discussion

This study concluded that there is a high (47%) in-hospital mortality in patients with cardiogenic shock of any etiology in our population and mortality risk did not vary significantly between sexes. Treatment approaches and procedures performed in our center were equitable and no sex disparities were observed.

Clinical evidence in this field is scarce. There are only few divergent observational studies addressing CS complicating AMI (3, 4) whose results cannot be extrapolated to any other different etiology. In addition, the proportion of ACS-related CS is significantly lower in women (9). In contrast, our cohort of patients includes a wide range of underlying conditions and provides valuable insight into this complex area.

Moreover, women are underrepresented, as the proportion of women included in previous series is relatively low (ranging from 25 to 45%) as it happened in our case. It is also remarkable that they are usually older and suffer from more comorbidities (9–11) which might be associated with a worse prognosis. Conversely, our cohort is quite balanced with respect to baseline characteristics and prognostic factors. However, it is remarkable that only 24% of our cohort are women. We hypothesize that this low percentage may reflect a low prevalence of severe heart disease at young ages in female patients, although a patient-selection bias cannot be ruled out.

Some authors suggest that management disparities may also play a crucial role in ACS-related CS (12). Vallabhajosyula et al. described that young women are treated less aggressively with coronary angiography and experience higher in-hospital mortality than men (13). On the other hand, this inequity seems to be less noticeable in patients requiring advanced support treatment. The use of temporary mechanical circulatory support, mechanical ventilation or renal replacement therapy was not significantly different in both groups in the vast majority of studies (14, 15).

The major limitation of our single-center study is its observational retrospective design, with a modest sample size, which makes necessary further validation in a prospective trial. It is of paramount importance to highlight that our results must be contextualized in a CS cohort of high severity and complexity, with a high accessibility to heart transplantation in an urgent code.

Noteworthy is also the modest ability of SCAI classification for predicting in-hospital mortality, similar in both sexes. To our knowledge, this fact has not been previously described in the literature. Despite the limitations mentioned above, we strongly believe that SCAI classification is easy to apply in clinical practice and provides useful baseline information about the prognosis of patients in CS.

## Conclusions

The management of cardiogenic shock remains a clinical challenge even in hub centers. Almost one in four patients in this series are women. In- hospital mortality risk is still high (47% in our population) and did not differ significantly between both sexes. Treatment approaches and procedures performed were equitable and no sex disparities were observed in our cohort. The yield of SCAI risk classification was only fair and similar in both genders.

## Data Availability Statement

The original contributions presented in the study are included in the article/supplementary material, further inquiries can be directed to the corresponding author/s.

## Ethics Statement

The studies involving human participants were reviewed and approved by Hospital Puerta de Hierro. Written informed consent for participation was not required for this study in accordance with the national legislation and the institutional requirements.

## Author Contributions

SL and RI wrote the manuscript and collaborated in data collection. FH and JS conceived of the presented idea and supervised the findings of this work. CM and MR developed the theory, performed the computations, and verified the analytical methods. SV, JE, JO, and JV verified the numerical results and support the results. All authors discussed the results and contributed to the final manuscript. All authors contributed to the article and approved the submitted version.

## Funding

This work was supported by Fundación Investigación Puerta de Hierro.

## Conflict of Interest

The authors declare that the research was conducted in the absence of any commercial or financial relationships that could be construed as a potential conflict of interest.

## Publisher's Note

All claims expressed in this article are solely those of the authors and do not necessarily represent those of their affiliated organizations, or those of the publisher, the editors and the reviewers. Any product that may be evaluated in this article, or claim that may be made by its manufacturer, is not guaranteed or endorsed by the publisher.

## Abbreviations

ACS: Acute coronary syndrome
AMI: Acute Myocardial infarction
AUC: Area under the curve
CHF: Chronic heart failure
COPD: Chronic obstructive pulmonary disease
CS: Cardiogenic shock
ECMO: venoarterial extracorporeal membrane oxygenation
LVAD: Left ventricular assist device
MCS: Mechanical circulatory support
ROC curves: Receiver operating characteristic
SCAI: Society of Cardiovascular Angiography and Interventions
VIS: Vasoactive inotropic score.
